# Mutations in glioblastoma proteins do not disrupt epitope presentation and recognition, maintaining a specific CD8 T cell immune response potential

**DOI:** 10.1038/s41598-024-67099-2

**Published:** 2024-07-19

**Authors:** Renata Fioravanti Tarabini, Gustavo Fioravanti Vieira, Maurício Menegatti Rigo, Ana Paula Duarte de Souza

**Affiliations:** 1 Laboratory of Clinical and Experimental Immunology, Infant Center, School of Health Science, Pontifical Catholic University of Rio Grande do Sul (PUCRS), Porto Alegre, Brazil; 2 Kavraki Lab, Department of Computer Science, Rice University, Houston, TX USA; 3 Post-Graduation Program in Genetics and Molecular Biology, Universidade Federal do Rio Grande do Sul, Porto Alegre, Brazil; 4 Post-Graduation Program in Health and Human Development, Universidade La Salle, Canoas, Brazil; 5 Present Address: Center for Discovery and Innovation, Hackensack Meridian Health, Nutley, NJ USA

**Keywords:** Cancer, Computational biology and bioinformatics, Immunology

## Abstract

Antigen-specific cytotoxic CD8 T cells are extremely effective in controlling tumor growth and have been the focus of immunotherapy approaches. We leverage in silico tools to investigate whether the occurrence of mutations in proteins previously described as immunogenic and highly expressed by glioblastoma multiforme (GBM), such as Epidermal Growth Factor Receptor (EGFR), Isocitrate Dehydrogenase 1 (IDH1), Phosphatase and Tensin homolog (PTEN) and Tumor Protein 53 (TP53), may be contributing to the differential presentation of immunogenic epitopes. We recovered Class I MHC binding information from wild-type and mutated proteins using the Immune Epitope Database (IEDB). After that, we built peptide-MHC (pMHC-I) models in HLA-arena, followed by hierarchical clustering analysis based on electrostatic surface features from each complex. We identified point mutations that are determinants for the presentation of a set of peptides from TP53 protein. We point to structural features in the pMHC-I complexes of wild-type and mutated peptides, which may play a role in the recognition of CD8 T cells. To further explore these features, we performed 100 ns molecular dynamics simulations for the peptide pairs (wt/mut) selected. In pursuit of novel therapeutic targets for GBM treatment, we selected peptides where our predictive results indicated that mutations would not disrupt epitope presentation, thereby maintaining a specific CD8 T cell immune response. These peptides hold potential for future GBM interventions, including peptide-based or mRNA vaccine development applications.

## Introduction

Glioblastoma multiforme (GBM) is the most common and aggressive form of tumor of the central nervous system (CNS) with poor prognosis and high levels of morbidity and mortality^1^. GBM incidence in adults is 3.7 per 100,000 person-years^2^, and only 2.2% of patients are estimated to survive three years or more after diagnosis^3^. In children, GBM accounts for approximately 8–12% of all primary CNS tumors, and about 25 percent of children with this tumor live for five years or more^4^. Unfortunately, therapeutic options for these tumors are still minimal.

GBM is characterized by intra- and intertumoral heterogeneity, highly invasive cellular properties, and an immunosuppressive microenvironment that promotes GBM growth through complex interactions^5^. Patients with GBM considered long-term survivors have more significant infiltration of CD8 T cells than short-term survivors, positively correlating CD8 T cells with a better survival rate^6^. Based on this knowledge, immunotherapy emerges as a promising therapeutic approach^7^.

T-cell-mediated immunotherapy has shown promise in clinical trials for cancer. The effectiveness of immunotherapy based on the T cell response depends on the stimulatory context and the adequate choice of tumor antigen to be used, more precisely on the T cell epitopes contained in these tumor proteins^8^. Peptides stably bound to MHC-I, also called epitopes, will be presented on the cell's surface for later recognition by the T-cell receptor (TCR). CD8 T cells that recognize these epitopes exert direct effector functions, producing inflammatory or regulatory cytokines and promoting cytotoxicity. Also, CD8 T cell response can generate long-term memory populations allowing the host to respond rapidly to subsequent encounters of the same epitope^9^.

There are several immunogenic CD8 T cell epitopes described for GBM, especially in the highly expressed proteins, such as Epidermal Growth Factor Receptor (EGFR), Isocitrate Dehydrogenase 1 (IDH1), Phosphatase and Tensin homolog (PTEN), and Tumor Protein 53 (TP53). However, tumor progression occurs regardless of the presence of T cell-mediated response, showing the high capacity of this type of tumor to escape from the immune surveillance mechanisms^10^ and induce immunosuppression. For instance, GBM can create a highly immunosuppressive microenvironment characterized by the presence of cells like regulatory T cells (Tregs), myeloid-derived suppressor cells (MDSCs), and M2-polarized macrophages^11^. The expression of specific checkpoint molecules (e.g., PD-L1) can also lead to T cell exhaustion and inhibition of T cell cytotoxic function^12^. Another important aspect is that the tumor proteins constantly mutate, which may affect the recognition of immunogenic epitopes^13,14^.

In this study, we focused on the occurrence of mutations in proteins previously described as immunogenic and highly expressed by GBM, such as TP53, PTEN, EGFR, and IDH1. We investigated the role of prominent mutations on the disappearance or differential presentation of immunogenic epitopes. We used the combined MHC-I intracellular pathway prediction tools from the Immune Epitope Database (IEDB)^15,16^, alongside machine learning (ML) methods for pMHC-I immunogenicity prediction^17^. Furthermore, we leverage HLA-Arena^18^ to create and execute workflows for structural modeling, analysis, and visualization of pMHC-I complexes. We identified six GBM peptides from TP53 protein whose presentation remains unaffected by mutations, as consistently predicted by in silico tools. To further validate these findings, we performed 100 ns of molecular dynamics simulation on these six peptide pairs (wt/mut). The simulation results supported the stability and binding potential of these peptides, reinforcing their promise as targets for the development of immunotherapeutic strategies in GBM treatment, potentially paving the way for more effective clinical interventions.

## Results

### Immunogenic epitopes containing missense mutations can still be generated through MHC-I pathway

We looked for missense mutations on the TCGA-GDC cancer database, and we found that the EGFR, IDH1, PTEN, and TP53 proteins were the most frequently mutated proteins in GBM. A total of 49, 5, 57, and 64 missense mutations were uncovered for EGFR, IDH1, PTEN, and TP53, respectively (Supplementary Table S1). From this list of mutations, 4 occurred in immunogenic epitopes of EGFR, 1 in IDH1, 2 in PTEN, and 22 in TP53 (Supplementary Table S2). Immunogenic epitopes were recovered from IEDB.

Next, we used the combined predictors of proteasomal processing, TAP transport, and MHC binding to score and identify the probability of presentation of the mutated epitopes in selected proteins. The total score for wild-type and mutated epitopes and their respective upstream/downstream epitopes were compared (Supplementary Figs. S1 and S2). Our analysis revealed no significant differences in the mean total scores of mutated epitopes compared to their respective wild-type counterparts. This suggests that the missense mutations do not impact the presentation of these epitopes. However, when directly comparing the pair of wild-type and mutated epitopes we found that in 39 pairs, the wild-type and mutated epitope total scores were below the mean total score, and the difference in the total score between the wild-type and the mutated epitope was higher than 0.5 (arbitrary value). Because this result suggests that the mutation might influence the peptide generation in the processing pathway, these 39 pairs were not considered for downstream analysis. We selected the 83 remaining pairs for further structural analysis.

### Structural analysis of MHCs in the context of wild-type and mutated epitopes

As explained in the Materials and Methods section, we kept the same MHCflurry cutoff value for both wild-type and mutant epitopes. Because HLA-Arena models only pMHC-I complexes that pass through MHCflurry cutoff, there were cases where the pair (wild-type/mutant) was not modeled (Supplementary Table S3). In our case, 24 pairs out of 83 were modeled in the context of different HLA molecules (Table 1) and used for downstream analyses.

**Table 1:** Epitopes with their respective alleles modeled through structural analysis using the HLA-Arena platform. The mutated amino acids are highlighted (missense) according to the GDC-Cancer.

Protein	HLA allele	MHCflurry cutoff (nM)	Wild-type	Mutated
IDH1	B*15:01	370	WVKPIIIGRHAY	WVKPIIIGHHAY–R132H
				WVKPIIIGGHAY–R132G
				WVKPIIIGCHAY–R132C
TP53	B*27:05	385	GRNSFEVRV	GRNSFEVCV–R273C
				GRNSFEVHV–R273H
	A*23:01	18	TYSPALNKMF	TYSPALNNMF–K132N
				TYSPAFNKMF–L130F
	A*24:02	39	TYSPALNKMF	TYSPALNNMF–K132N
				TYSPAFNKMF–L130F
				TYYPALNKMF–S127Y
	A*02:01	172	GLAPPQHLIRV	GLAPPQHLTRV–I195T
	B*07:02	25	RPILTIITL	RPILTSITL–I254S
				RPILTISTL–I255S
	A*02:01	39	LLGRNSFEV	LLVRNSFEV–G266V
				LLGPNSFEV–R267P
				LLGWNSFEV–R267W
	A*23:01	115	EYLDDRNTF	EYLDDRNIF–T211I
	A*02:01	635	ALNKMFCQL	ALNNMFCQL–K132N
				ALNKMFCEL–Q136E
	A*02:06	324	ALNKMFCQL	ALNNMFCQL–K132N
	A*68:02	21	STPPPGTRV	STPPPGNRV–T155N
	B*57:01	19	LAKTCPVQLW	LTKTCPVQLW–A138T
	B*51:01	752	DGLAPPQHLI	DGLALPQHLI–P190L
	A*02:06	119	KMFCQLAKT	KMFCELAKT–Q136E

**Figure 1:**
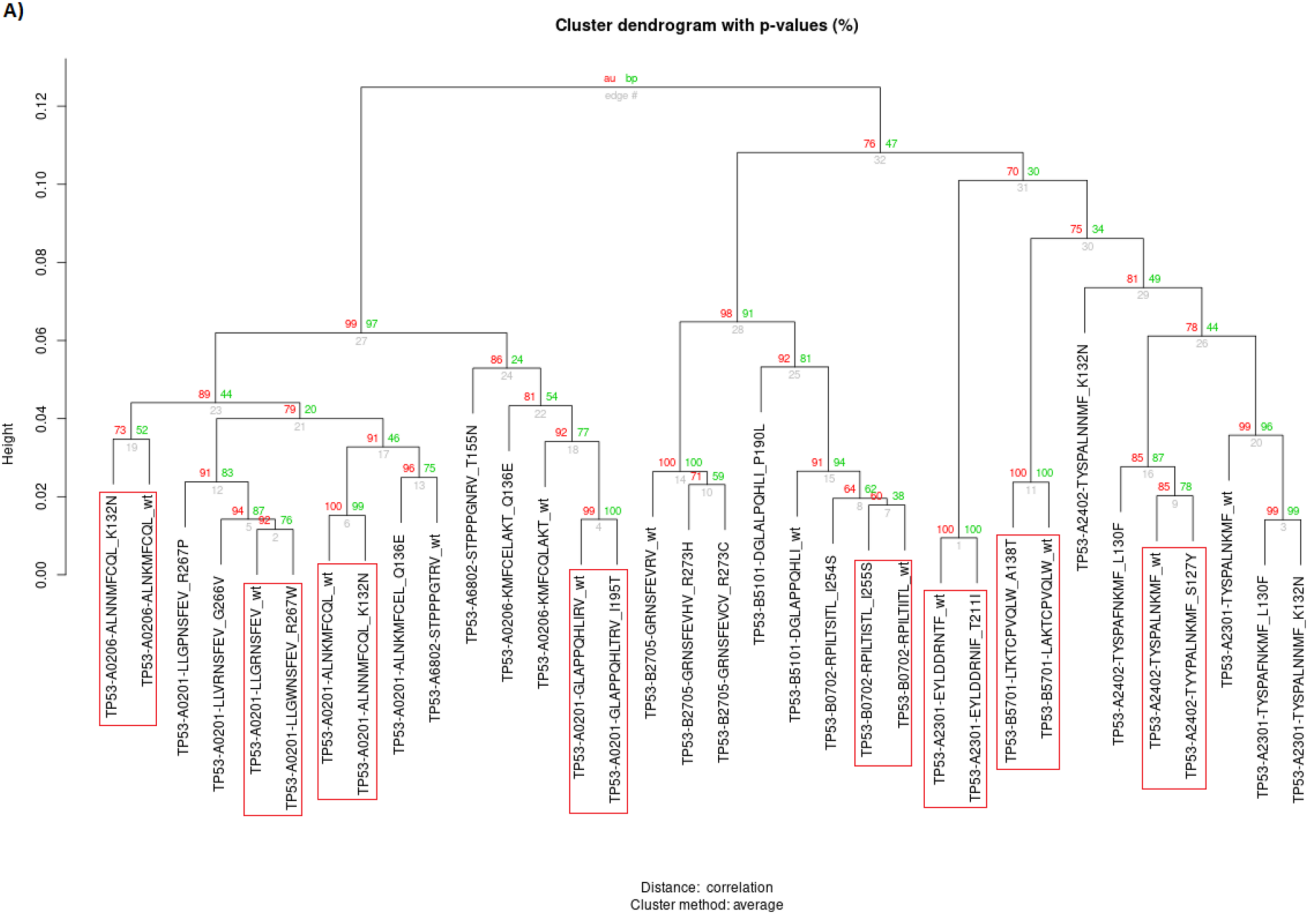

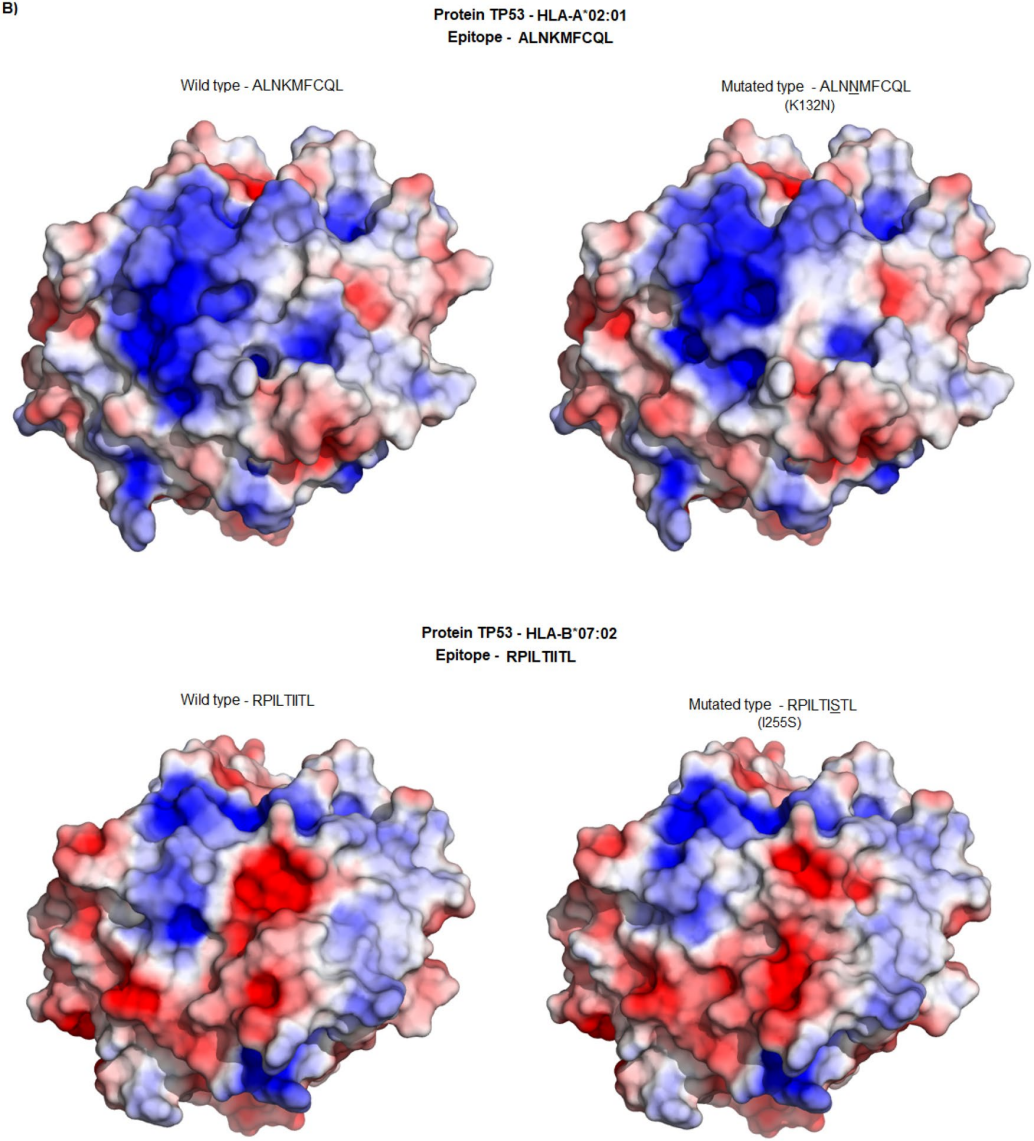
HCA of the electrostatic potential and exemplification of their electrostatic and structural similarity. (**A**) Dendrogram of the TP53 protein demonstrating the pairs of pMHC complexes, the red rectangles indicate the pairs preserving structural characteristics. (**B**) Top view of pMHC complexes highlighting the electrostatic similarity between wild-type and mutated epitopes from TP53 protein (red, white, and blue represent negative, neutral, and positive charges, respectively). AU, Approximately Unbiased; BP, Bootstrap Probability.

To evaluate the similarity of the TCR-interacting surface of the remaining 24 pMHC-I complex pairs, we performed a hierarchical clustering analysis (HCA) of the electrostatic potential collected from 46 regions of interest (Supplementary Fig. S3). The use of HCA for selection of similar pMHC-I pairs based on electrostatic features was validated elsewhere^19–21^. Figure 1A shows the HCA for TP53-derived peptides, while Fig. 1B illustrates the image similarity between two distinct peptide-MHC class I (pMHC-I) pairs (wild-type/mutant). Although qualitative differences and similarities in the pMHC-I complexes can be observed, the HCA offers a quantitative approach to emphasize these characteristics more distinctly, at the same time allowing the clustering of similar pairs. For TP53 epitopes, 8 pairs of pMHC complexes were clustered together.

**Figure 2:**
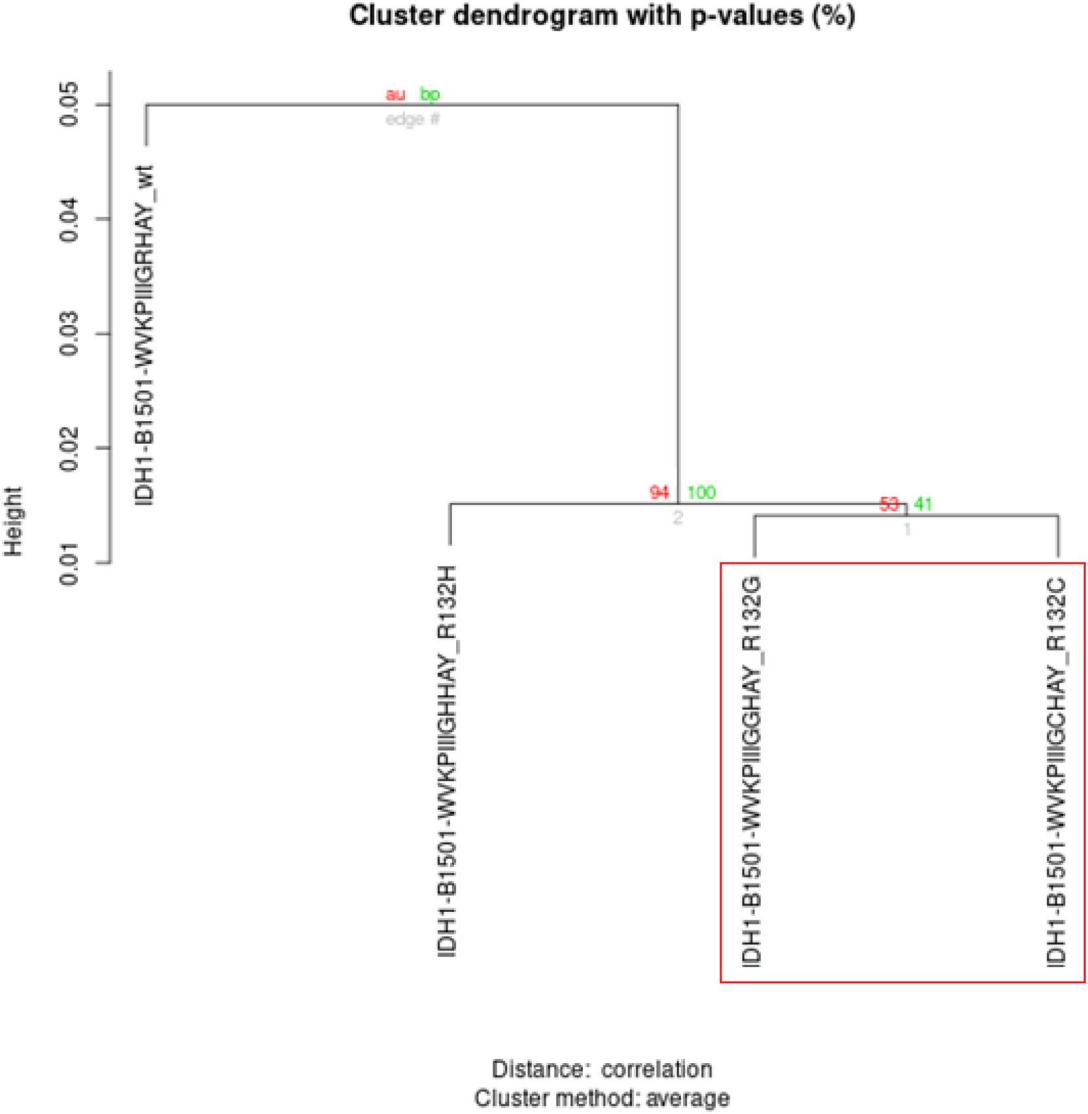
Dendrogram illustrating the HCA of the IDH1 protein, focusing on four pMHC complexes: WVKPIIIGRHAY-WT, WVKPIIIGHHAY-R132H, WVKPIIIGGHAY-R132G, and WVKPIIIGCHAY-R132C. The analysis reveals distinct clustering, with the mutated pMHC complexes R132G and R132C forming one cluster, followed by the appearance of R132H. The wild-type (WT) complex is positioned as the most distant group, highlighting the significant variance from the mutated forms.

The HCA results for IDH1 proteins showed a different pattern, positioning the wild-type epitope on a distant branch compared to the mutated epitopes. Since we are interested in the pair of epitopes where the wild-type is structurally similar to the mutated counterpart, we did not pursue additional analysis on IDH1 epitopes (Fig. 2).

### Filtering targeted epitopes for GBM therapy

To pursue new targets for GBM therapy, we selected only the epitope pairs that presented better-predicted results in all analyses (i.e., sequence-based prediction and HCA–based structural analysis). The rationale behind this approach is to select epitopes where the described mutation has a lower chance of affecting the CD8 T cell-specific immune response. In total, six TP53 epitopes stand out (Table 2). Since immunogenicity is crucial, we submitted each pMHC-I to an immunogenicity prediction tool (TLImm)^17^. We observed that the scores were similar to or even better (3 out of 6 cases) than the well-known immunogenic wild-type peptide.

**Table 2:** Pairs of epitopes were selected due to their better-predicted results in all analyses (i.e., processing pathway and presentation of MHC-I antigens, modeling of pMHC-I complexes, and hierarchical clustering).

Protein	Allele	Peptides (wt/mut)	Total score (wild/mutated)	Clustering (AU/BP)	TLImm score
TP53	A*02:01	ALNKMFCQL	− 0.09	100/99	0.462
		ALNNMFCQL (K132N)	0.27		0.485
	A*02:01	LLGRNSFEV	0.11	92/76	0.520
		LLGWNSFEV (R267W)	0.38		0.454
	A*23:01	EYLDDRNTF	0.27	100/100	0.445
		EYLDDRNIF (T211I)	− 0.1		0.365
	A*24:02	TYSPALNKMF	1.08	85/78	0.607
		TYYPALNKMF (S127Y)	1.39		0.569
	B*07:02	RPILTIITL	0.9	60/38	0.318
		RPILTISTL (I255S)	1.27		0.463
	B*57:01	LAKTCPVQLW	0.16	100/100	0.441
		LTKTCPVQLW (A138T)	0.22		0.454

Immunogenicity prediction score from TLImm is also shown.

To further explore structural features in a dynamic environment, we decided to run 100 ns molecular dynamics simulation for all 12 pMHC-I complexes selected from Table 2. We extracted the main quantitative features from each simulation, such as Root Mean Square Deviation (RMSD), Root Mean Square Fluctuation (RMSF), Radius of Gyration (RoG), and mean contacts between the epitope and the MHC-I (Table 3).

**Table 3:** Epitopes resulting from the prediction showed a strong binding affinity for MHC-II.

HLA allele	Epitope pair	Average ± SD Protein RMSD (nm)	RMSD effect size (Cohen’s d)	Cohen’s d descriptor	Average ± SD epitope RMSD (nm)	RMSD effect size (Cohen’s d)	Cohen’s d descriptor	Average ± SD Epitope RMSF (nm)	Average ± SD RoG (nm)	Mean contacts (< 0.4 nm)
A*02:01	ALNKMFCQL	0.297 ± 0.03	1.920	Very large (WT higher)	0.26 ± 0.03	0.140	Small (WT higher)	0.119 ± 0.03	1.75 ± 0.008	44.9
	ALNNMFCQL	0.244 ± 0.02			0.255 ± 0.03			0.141 ± 0.03	1.73 ± 0.007	25.2
A*02:01	LLGRNSFEV	0.228 ± 0.02	− 1.369	Very large (mut higher)	0.308 ± 0.06	2.000	Huge (WT higher)	0.18 ± 0.05	1.72 ± 0.006	32.6
	LLGWNSFEV	0.271 ± 0.04			0.207 ± 0.02			0.1 ± 0.03	1.74 ± 0.01	62.5
A*23:01	EYLDDRNTF	0.245 ± 0.02	0.653	Medium (WT higher)	0.192 ± 0.01	− 2.200	Huge (mut higher)	0.08 ± 0.02	1.72 ± 0.006	76.1
	EYLDDRNIF	0.233 ± 0.02			0.235 ± 0.02			0.09 ± 0.04	1.72 ± 0.007	83.1
A*24:02	TYSPALNKMF	0.345 ± 0.05	2.782	Huge (WT higher)	0.386 ± 0.08	1.600	Very large (WT higher)	0.171 ± 0.04	1.74 ± 0.01	24.3
	TYYPALNKMF	0.242 ± 0.02			0.279 ± 0.05			0.149 ± 0.06	1.71 ± 0.007	130.1
B*07:02	RPILTIITL	0.329 ± 0.04	2.084	Huge (WT higher)	0.212 ± 0.02	− 2.400	Huge (mut higher)	0.08 ± 0.02	1.76 ± 0.02	57.8
	RPILTISTL	0.256 ± 0.03			0.376 ± 0.09			0.246 ± 0.07	1.72 ± 0.01	43.8
B*57:01	LAKTCPVQLW	0.244 ± 0.03	− 1.562	Very large (mut higher)	0.313 ± 0.03	0.080	Very small (WT higher)	0.157 ± 0.04	1.72 ± 0.01	12.6
	LTKTCPVQLW	0.295 ± 0.03			0.31 ± 0.05			0.131 ± 0.04	1.74 ± 0.01	52.5

Strong binder threshold (% Rank): 2.00.

**Figure 3:**
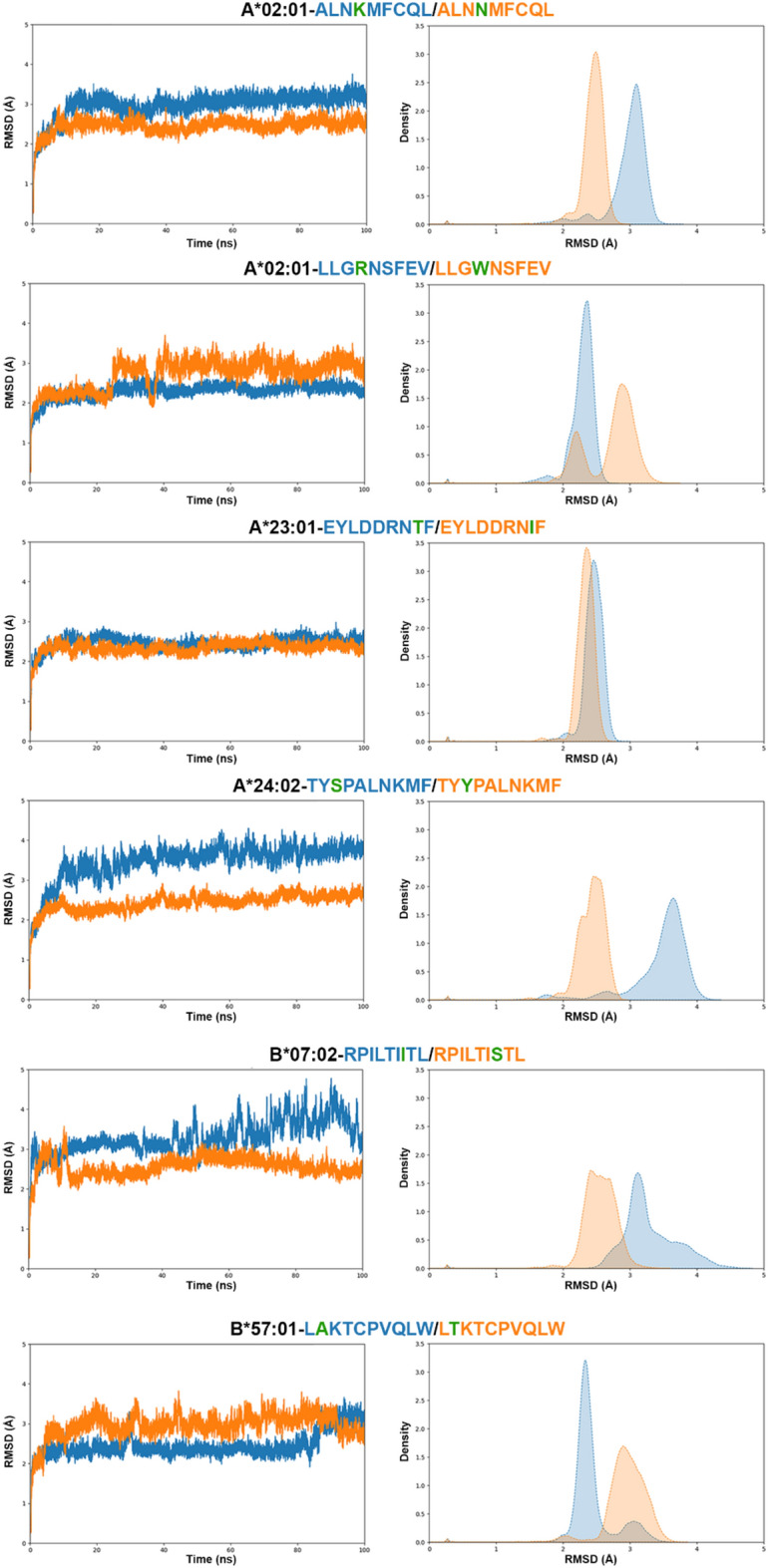
Protein RMSD (in Å) for 6 selected pMHC-I pairs. The graphs on the left show the RMSD along time (in nanoseconds), while the graphs on the right shows the RMSD density plot for the whole simulation. Wild-type peptides are colored in blue, while the mutated counterpart is colored in orange. The mutated residue is shown in green.

One of the measures reflecting pMHC-I stability is the RMSD. In most cases, the average RMSD for both the protein and epitope was lower for the mutated peptide (Table 3). The effect size of this difference, quantified using Cohen’s d descriptor, ranged from medium to huge (see details in the Methods section). In all simulations, the RMSD reached a plateau before the 100 ns mark, indicating that the simulation time was appropriate (Fig. 3). Interestingly, the density plot analysis of the protein RMSD revealed primarily single peaks, except for the HLA-A*02:01-LLGWNSFEV complex, which displayed two distinct peaks. When we analyzed the epitope RMSD we could observe that more pMHC-I complexes showed multiple peaks, likely because the calculation involved only 9 to 10 residues (Supplementary Fig. S4). Nonetheless, the epitope RMSD remained stable throughout the simulation in all cases.

**Figure 4:**
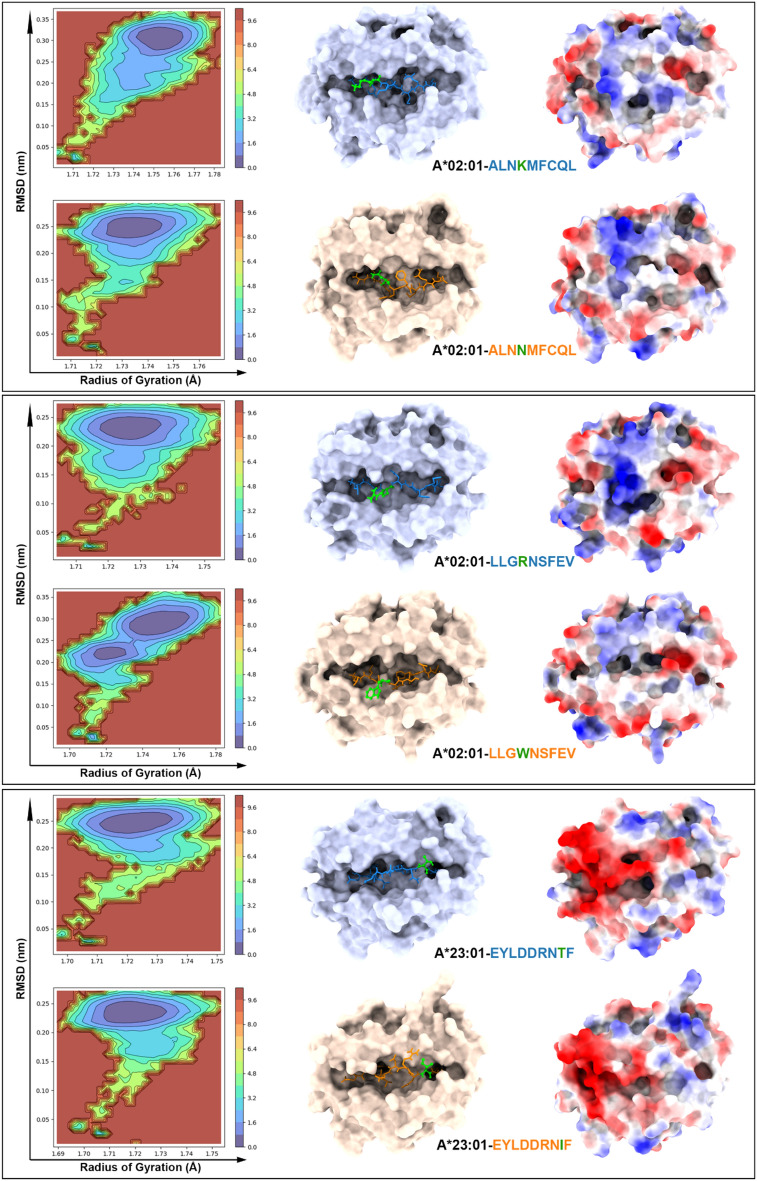
Free energy surface (FES) landscape (left) and lowest energy structures retrieved from simulation (right) for complexes HLA-A*02:01-ALNKMFCQL/ALNNMFCQL, HLA-A*02:01-LLGRNSFEV/LLGWNSFEV, and HLA-A*23:01-EYLDDRNTF/EYLDDRNIF. The FES plots depict the stability and conformational changes of the peptide-MHC complexes. In the middle, the MHC structures are shown with the peptide atoms highlighted, where the mutated residues are marked in green. On the right, the electrostatic potential surface of the peptide-MHC (pMHC-I) complex is displayed, illustrating the potential impact of mutations on the complex's surface properties.

**Figure 5:**
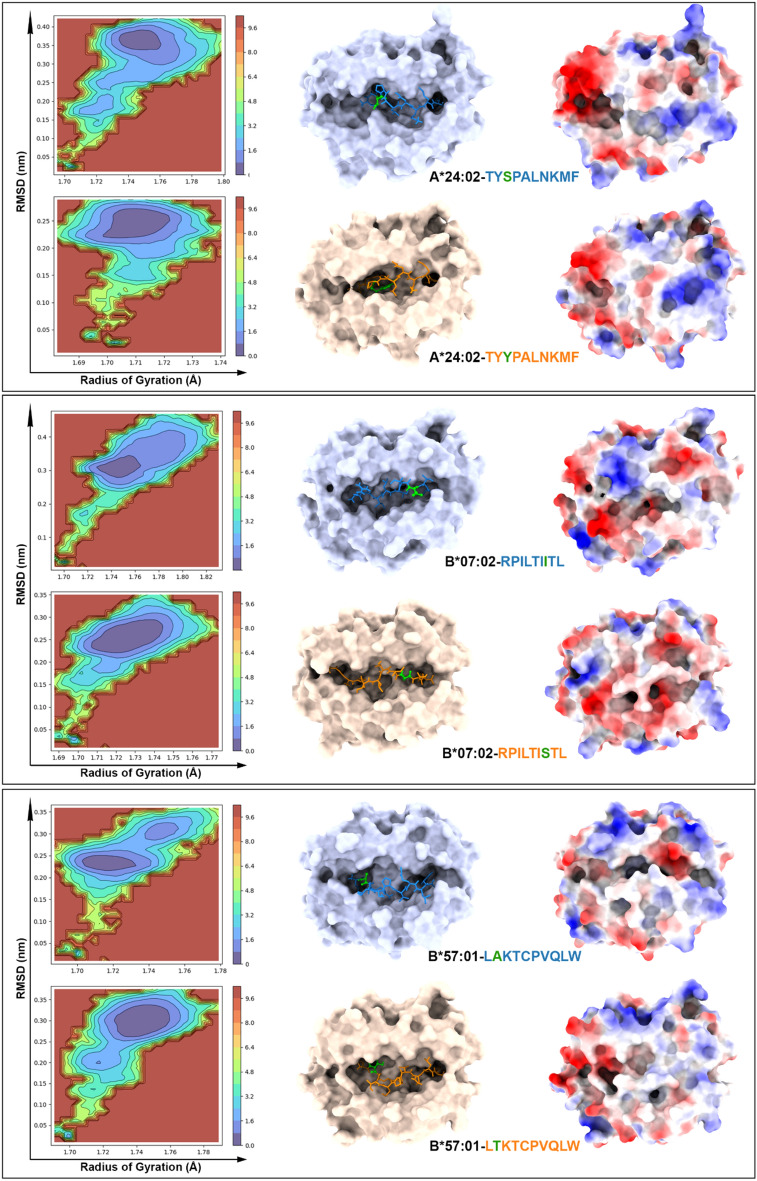
Free energy surface (FES) landscape (left) and lowest energy structures retrieved from simulation (right) for complexes HLA-A*24:02-TYSPALNKMF/TYYPALNKMF, HLA-B*07:02-RPILTIITL/RPILTISTL, and HLA-B*57:01-LAKTCPVQLW/LTKTCPVQLW. The FES plots depict the stability and conformational changes of the peptide-MHC complexes. In the middle, the MHC structures are shown with the peptide atoms highlighted, where the mutated residues are marked in green. On the right, the electrostatic potential surface of the peptide-MHC (pMHC-I) complex is displayed, illustrating the potential impact of mutations on the complex's surface properties.

We were also interested in evaluating the free energy surface (FES) landscape for each of the pairs analyzed (Figs. 4 and 5). The FES can be used to understand the stability and conformational changes of molecular systems because it is derived from RMSD and RoG values. Complexes HLA-A*02:01-ALNKMFCQL/ALNNMFCQL and HLA-A*23:01-EYLDDRNTF/EYLDDRNIF show a slightly different distribution of energy minima, but FES plots support the hypothesis that the mutation in these cases does not drastically destabilize the peptide-MHC complex. Other complexes show a more complex scenario, indicating that the mutation can introduce alternative conformations (e.g., HLA-A*02:01-LLGRNSFEV/LLGWNSFEV, which aligns with the protein RMSD plots) or additional flexibility (e.g., HLA-B*07:02-ALNKMFCQL/ALNNMFCQL, consistent with the epitope RMSF values in Table 3). Still, and supported by RMSD analysis, the mutation was not sufficient to destabilize any of the pMHC complexes analyzed.

We sought to determine if the electrostatic potential would change when comparing the modeled pMHC-I models against the lowest energy structures generated during the molecular dynamics (MD) simulation (Supplementary Fig. S5), and we also compared the lowest energy structures to the wild-type/mutated pairs. We observed primarily topographical modifications, along with differences in the distribution of charges around the TCR-interacting surface for some of the pMHC-I complexes. For instance, in the HLA-A*02:01-LLGRNSFEV/LLGWNSFEV complex, the substitution of Arginine with Tryptophan resulted in a loss of positive charges (Fig. 4). In the HLA-B*07:02-RPILTIITL/RPILTISTL complex, despite the modification occurring at the C-terminus of the epitope, a new positive charge emerged at the N-terminal region near residue 2 (Fig. 5). Additionally, the HLA-B*57:01-LAKTCPVQLW/LTKTCPVQLW complex exhibited significant topographical changes, likely due to the need to accommodate the more hydrophilic Threonine residue in the 10-mer peptide (Fig. 5). This adaptation resulted in a pronounced negative charge at the N-terminal part of the epitope and a gradual loss of the negative charge at the C-terminus.

**Figure 6:**
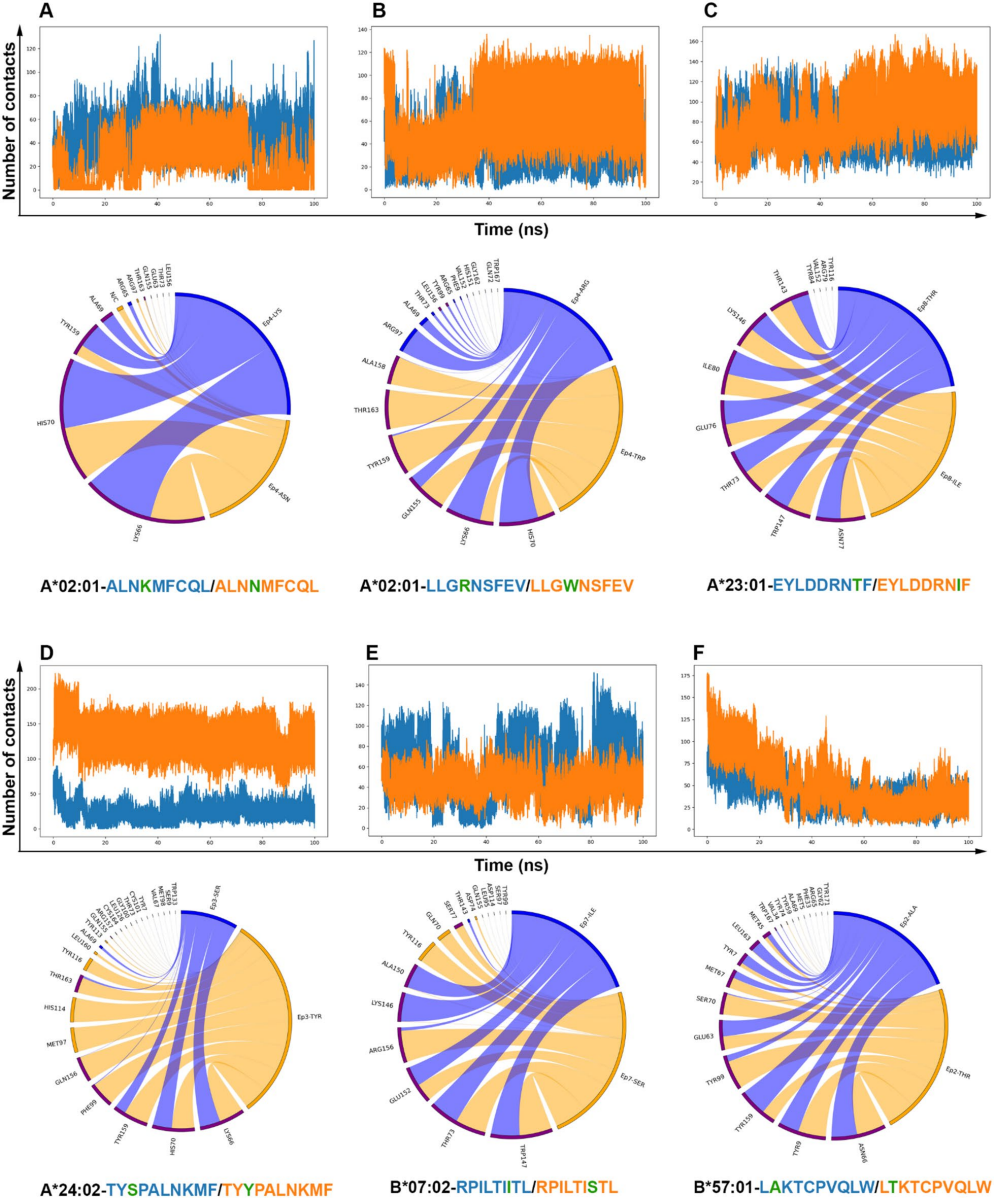
Contacts performed in the range of 0.4 nm between peptide and MHC-I. The line graphs represent the number of contacts performed along the simulation (wild-type peptide in blue and mutated peptide in orange). The circular alluvial plots show the residues interacting with the wt/mutated residue. The links are in blue (wt residue) or orange (mut residue). In each sector, the color blue, orange, or purple represent the MHC residues that are being contacted by the wt residue, by the mut residue or by both, respectively.

Finally, we aimed to gain a better understanding of the contact map during the simulation between the peptide (wild-type/mutant) and the respective MHC-I (Fig. 6). We focused on contacts within the range of 0.4 nm (4 Å), a distance that captures key interactions such as hydrogen bonds, van der Waals interactions, hydrophobic contacts, and potential salt bridges. These interactions are crucial for the stability and specificity of the peptide-MHC-I complex and can provide insights into the effects of mutations on peptide binding and presentation.

We observed that the contacts tended to increase or remain stable in most of the pMHC-I complexes, with the exceptions illustrated in Fig. 6A,F. It is clear that most of the time, the same MHC-I residues were contacted by both wild-type and mutated residues throughout the simulation. However, in some specific cases, like the HLA-A*02:01-LLGRNSFEV/LLGWNSFEV complex (Fig. 6B), MHC-I residues were mainly contacted by the mut residue. One particular noteworthy case is the HLA-A*24:02-TYSPALNKMF/TYYPALNKMF complex (Fig. 6D). In this case, the number of contacts between the mutated epitope and the MHC-I was five times greater than those of the wild-type peptide. This significant increase suggests that the mutation greatly enhances the interaction between the epitope and the MHC-I. Additionally, some key contacts were unique to the mutated epitope and MHC-I, involving residues such as HIS114 and MET97, which were not observed in the wild-type interactions. These unique contacts may positively contribute to binding affinity and specificity observed in the mutated complex.

### MHC-II binding analysis of the selected epitopes

Because of the importance of CD4 T cells in helping CD8 T cells and exerting antitumor immune response^22^, we used a sequence-based MHC-II peptide predictor (https://services.healthtech.dtu.dk/services/NetMHCII-2.3/) to rank the binding capacity of the epitopes selected on Table 3. As a result, we obtained 9 epitopes (wild-type and mutated) that showed strong binding affinity to the alleles described for MHC-II (Table 3). Alleles were selected according to epitope prediction based on HLA class II binding in the human population^23^. These data indicate that the selected epitopes can also bind MHC-II inducing a CD4 T cell response.

## Discussion

In the present study we performed a computational analysis to highlight the role of mutations in different GBM-related proteins and the peptide presentation in the context of MHC-I receptors. We showed that despite the presence of mutation in these peptides, pMHC-I structural similarity may still elicit the same CD8 T cell response. This conclusion is supported by molecular dynamics experiments.

First, we selected 83 mutant and wild-type peptide pairs based on MHC-I pathway prediction tools results, indicating that MHC-I presentation of mutated epitopes on selected proteins may be unaffected. After adjusting MHC-I binding cutoffs, 24 from 83 pMHC-I complex pairs were modeled. Applying the hierarchical cluster analysis on TCR-interacting surface features of each pMHC-I complex, epitopes from TP53 protein presented better clustering. Finally, we selected 6 pairs of putative epitopes based on the results of all the computational tools used. Our data suggest that these selected putative epitopes can be future targets for the development of new therapies against GBM, given that the mutation that will occur in the epitope might not affect the CD8 T cell response. Also, four of these pairs can also bind the MHC-II and feasibly contribute to the CD4 T cell response.

To determine if the observed differences in RMSD, and consequently stability, were significant for cases where the mutated epitope exhibited lower RMSD values, we employed both t-test statistics and Cohen’s d descriptor. The t-test results were significant (p < 0.001) in all cases, confirming the statistical significance of the differences. Furthermore, the effect sizes, as measured by Cohen’s d, were significant across all cases. These findings suggest that the mutated epitopes have a high likelihood of remaining stable on the cell surface when bound to MHC-I. Note that the proteins EGFR, IDH1 and PTEN, despite being highly expressed in GBM and with a high frequency of mutations, did not show a good overall result. This may be related to the number of mutations that are found in the immunogenic peptides. The majority of mutations in immunogenic epitopes occurred on TP53, 22 in total. It is not surprising that the TP53 protein is highlighted, since it is one of the most commonly dysregulated proteins in cancer, in 84% of GBM according to the TCGA and in up to 94.1% of GBM cell lines^24^. Also, TP53 is a well-known antigen recognized by antitumor immune response^25^. For example, Kim et al*.*, sequenced the entire exome in 163 patients with solid metastatic cancers, identified 78 who had missense mutations in TP53 and, through immunological screening, identified 21 unique T cell reactivity^26^. Certain mutations in this protein give rise to tumor-specific amino acid sequences that can provide T cell targets in the context of MHC-I^27^, although some mutations can lead to the impairment of already immunogenic epitopes.

Peptide-based vaccines against GBM have been already studied using different antigen targets^28^. There was a clinical trial of a personalized vaccine using four different peptides in relapsed GBM patients, but the trial did not meet the primary or secondary outcomes^29^. Although these results were not satisfactory, more targets for peptide vaccines are worth exploring^30^.A strategy to increase the binding to the MHC and improve peptide vaccines is the modification of antigenic peptide's primary anchors^31^. Borbulevych et al*.* demonstrated that the greater immunogenicity of the peptide is due to the greater stability of the pMHC complex, validating the anchor fixation approach to generate therapeutic candidate vaccines^32^. Here, for instance, we show a pMHC-I complex where we changed a key anchor position (B*57:01, position 2), from Alanine to Threonine, but this did not seem to affect the total number of contacts between peptide and MHC-I. Other studies have shown that modified TCRs can be used to produce T lymphocyte populations with the high specificity for use in antigen-specific T cell therapy^33^. Here, we demonstrate how different mutations influence the topography and electrostatic potential of the TCR-interacting surface features. This type of study provides insights into the structural characteristics of pMHC-I, which are crucial for advancing TCR engineering and enhancing our understanding of TCR-pMHC-I interactions. Mutations in the peptide-binding pockets of HLA-A2.1 also have significant effects on CD8 T cell recognition^34^.

One of the limitations of our study is the lack of immunogenicity evaluation of the selected mutated peptides using in vitro and in vivo assays for MHC-I binding and T-cell stimulation. Despite other studies have confirmed that in silico structural analysis can confirm immunogenicity of the peptides^20,21,35^, we also ran an immunogenicity predictor based on transfer learning called TLImm. The scores obtained show similar or, for some cases, better scores when comparing the mutated peptides to the wild-type, immunogenic ones.

In conclusion, our findings suggest that the complex presenting the mutated peptide TYYPALNKMF, when bound to HLA-A*24:02, exhibits the most promising characteristics for use as a GBM peptide target. Notably, the protein and epitope RMSD values for the mutant complex were significantly lower compared to those of the wild-type. Additionally, the FES plots and the electrostatic potential at the TCR-interacting surface were remarkably similar between the wild-type and mutated complexes. Finally, the mutated epitope demonstrated more than a fivefold increase in the number of contacts relative to the wild-type. These attributes underscore the potential of the TYYPALNKMF peptide as a viable target for GBM immunotherapy.

In summary, we report that through predictive analyses, we were able to discern mutations capable of influencing, or not, the immune response, thereby allowing the targeting of mutations that elicited a response for use in immunotherapeutic approaches. Based on the information outlined in this study, we propose potential targets for glioblastoma multiforme (GBM) therapies, all derived from the TP53 protein.

## Materials and methods

### Search for mutations in highly expressed glioma proteins and potentially immunogenic epitopes

We first accessed the TCGA Database (https://cancergenome.nih.gov/), which provides the main genomic alterations in different types of cancer obtained from patient samples, to search for genes in the GDC (Genomic Data Commons) portal. We aimed at genes that have a high frequency of missense mutations in GBM (https://portal.gdc.cancer.gov/): *EGFR*, *IDH1*, *PTEN*, and *TP53*. We used the IEDB Database to search for immunogenic epitopes present in these proteins, using the search filters “T Cell assay”, “class I MHC restriction”, “human host”, and “antigen”, in this way we selected only the epitopes capable of generating a T-cell response.

### Download the protein sequences

The sequences of *Homo sapiens* EGFR (UniProt ID: P00533), IDH1 (UniProt ID: O75874), PTEN (UniProt ID: P60484), and TP53 (UniProt ID: P04637) proteins were obtained from the UniProtKB database (http://www.uniprot.org/) in the FASTA format. We mutated the sequences manually according to the mutations found in the GDC^28,36–44^.

### Class I MHC antigen processing analysis

We next used the combined prediction tool “Proteasomal cleavage/TAP transport/MHC class I combined predictor” available on the IEDB platform. The FASTA sequences of wild-type and mutated proteins were used as input. As output, we got a list of peptides that could be presented at the cell surface with their respective results for Total Score (data not shown). From this list, we searched for the wild-type epitopes and the mutated epitopes previously selected from GDC. In addition to these, we also searched for epitopes downstream and upstream including the mutated amino acid position, thus covering all peptides containing that mutation. In this work, we focus on total score results because it combines proteasomal cleavage, TAP transport, and MHC binding predictions. We used the GraphPad Prism 9.4.0 software (GraphPad Software, Inc., San Diego, CA) to compare the total score mean from wild-type and mutated epitopes found for the same MHC-I allele as well as their upstream and downstream epitopes and perform pairwise statistical analysis (t-test; p < 0.05). To select the epitope pairs (wild-type and mutated) for further analysis, we defined the following rules:

(i) the described wild-type and corresponding mutated epitope should be above the average total score;
(ii) the difference in the total score between the wild-type and the mutated epitope is not higher than 0.5 (arbitrary value).

To analyze the immunogenicity probabilities, we used a transfer learning-based prediction tool for peptide immunogenicity (TLImm) (available at: https://github.com/KavrakiLab/TL-MHC/tree/master/TLImm). This tool leverages comprehensive datasets encompassing binding affinity and mass spectrometry (MS).

### Modeling of pMHC-I

We first used the MHCflurry tool^45^, included in the HLA-Arena, which based on the chosen cutoff, generates the predictions (represents the affinity to MHC-I binding) relative to each wild-type epitope previously selected. Then we set the HLA-Arena with this cutoff value and used the virtual screening notebook with default options to construct the pMHC-I complexes for each pair of wild-type and mutated epitopes for the corresponding MHC-I allele. We submitted peptides for HLA-Arena analysis with different lengths, since MHCflurry allows us to analyze peptides of up to 15 amino acids in length.

### Generation and visualization of pMHC-I electrostatic potential

We used the PyMOL program^46^ to generate a visual representation of the electrostatic potential of the TCR-interacting surface of each pMHC-I complex obtained from HLA-Arena. We aligned the pMHCs and obtained the RMSD values before imaging the electrostatic surface of each complex. We generated an in-house plugin (“histogram2csv'' available at https://github.com/LAD-PUCRS/Arena_SARS-BCG) to extract data regarding the mean and standard deviation of the RGB values of each image in 46 regions of interest (Supporting Information, Fig. S5) as described in^19^. These values were used for further hierarchical clustering analysis with the 'pvclust' package^47^ from R software (https://www.r-project.org/)^48^. Pvclust is designed to evaluate the uncertainty associated with hierarchical cluster analysis. Pvclust provides two types of p-values: AU (Approximately Unbiased) p-value and BP (Bootstrap Probability) value. The AU p-value, computed through multiscale bootstrap resampling, offers a more precise approximation to an unbiased p-value than the BP value, which is calculated using normal bootstrap resampling^47^. The input data are color histogram (RGB) values for each pMHC-I complex, regarding the information on charge distribution and surface structure. We followed the steps described at “https://github.com/LAD-PUCRS/Arena_SARS-BCG” to generate the dendrogram from the hierarchical analysis. These dendrograms depict pMHC-I electrostatic similarity based on RGB values. For the generation of electrostatic potential for the pMHC-I complexes derived from the molecular dynamics simulation we used ChimeraX v1.7^49^ with default parameters.

**Figure 7:**
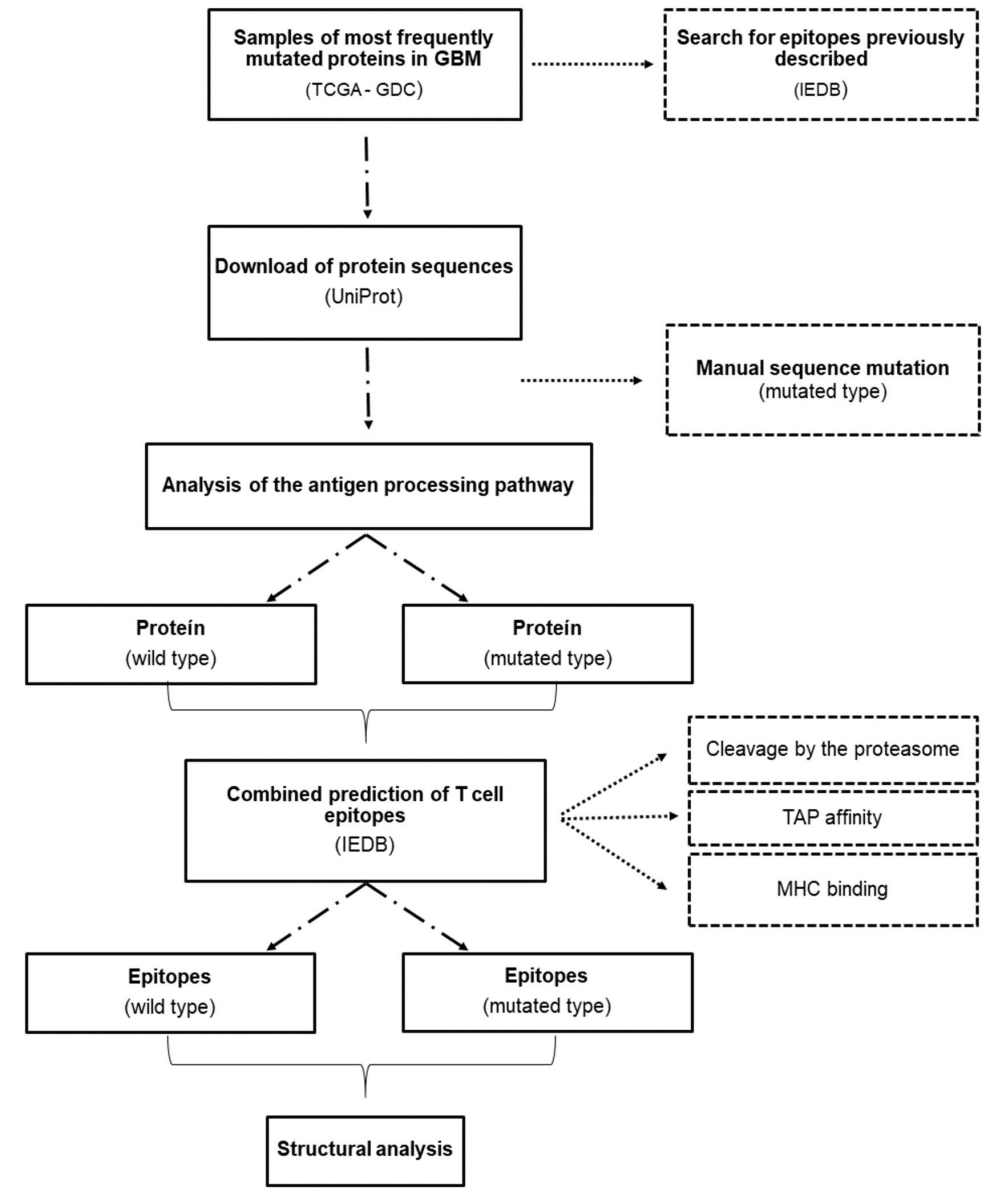
Flowchart with all the steps from data acquisition to processing and structural analysis of MHC-I complexes.

The summary of the methods is schematized on the flowchart (Fig. 7).

### Molecular dynamics simulations

#### Energy minimization, equilibration, and production

Input structures for molecular dynamics (MD) simulation were pre-processed with the PDB2PQR webserver^50^, fixed as needed and protonated at pH 7.0 using PROPKA algorithm^51^. To improve performance, the HLA receptor was truncated at residue 182, as previously described in Abella et al., 2020^52^. This resulted in a system composed by the domains alpha-1 and alpha-2 of the HLA and the bound peptide. The molecular dynamics (MD) simulations were performed with GROMACS 2024.2, CHARMM36 force field, and TIP3 water model. The simulation box was defined with a minimum distance of 1.5 nm (15 Å) between the protein and the box edge. The system was then solvated, and ions were added to neutralize the system and achieve a physiological ion concentration of 0.15 M NaCl. The algorithms v-rescale (tau-t = 0.1 ps) and Parrinello-Rahman (tau-p = 2 ps) were used for temperature and pressure coupling, respectively. A cutoff value of 1.2 nm was used for both the van der Waals and Coulomb interactions, with Fast Particle Mesh Ewald electrostatics (PME). The production stage of each MD simulation was preceded by three steps of energy minimization (EM) and eight steps of equilibration (EQ), as previously described^53^. Briefly, energy minimization (EM) was performed using the steepest-descent algorithm with position restraints applied to all heavy atoms of the amino acids, set at 5000 kJ mol^−1^ nm^−2^. The second EM step employed the same algorithm but removed the restraints. In the third EM step, the conjugate-gradient algorithm was used without any restraints to further relax the protein structure. The equilibration phase begins at a temperature of 310 K, maintained for 300 ps, with position restraints applied to the protein heavy atoms (5000 kJ mol^−1^ nm^−2^). This step allows solvation layers to form without disturbing the HLA-I folding. Subsequently, the temperature is reduced to 280 K, and the position restraints are gradually decreased. The temperature is then progressively increased to 300 K. These equilibration steps constitute the first 500 ps of each MD simulation. During the production stage, the temperature is kept constant at 300 K. Each pMHC-I complex was simulated for 100 ns.

#### Analysis

The raw trajectory files were post-processed using GROMACS tools (e.g., gmx trjconv) to perform rotational and translational alignment of sampled conformations, correct for the effects of periodic boundary conditions, and remove water molecules. The RMSD, RMSF, and Radius of Gyration were computed using the programs gmx rms, gmx rmsf, and gmx gyrate, respectively. For each pair, we performed a t-test to determine if there was a difference between WT and mutant RMSD values. A large absolute value indicates a large difference between the group means, while the sign (positive or negative) indicates the direction of the difference. In all cases (6 pairs analyzed), the RMSD frequency was statistically different between pairs. To evaluate the effect size, we computed Cohen’s d value for each pair (WT vs mutated), which is often used in the context of t-tests. As suggested by Cohen and expanded by Sawilowsky^54,55^, the effect size can be described as very small (d = 0.01), small (d = 0.20), medium (d = 0.50), large (d = 0.80), very large (d = 1.20), and huge (d = 2.0). The contact analysis was performed using the program gmx mindist, with a cutoff of 0.4 nm. Plots were generated using python in-house scripts and ggplot. The free energy surface (FES) was computed using the fes.py script, developed by Birgit Strodell from the Multiscale Modelling Group (http://www.strodel.info/) and optimized by Cristóvão Freitas Iglesias Junior (http://lmdm.biof.ufrj.br/). We further adapted the script to a new Python language (Python 3) and included new code to retrieve the lowest energy structure.

### Supplementary Information

Supplementary Information.

## Data Availability

The datasets generated during and/or analyzed during the current study are available from the corresponding author on reasonable request.
