# Supplementary material for: Mutations in glioblastoma proteins do not disrupt epitope presentation and recognition, maintaining a specific CD8 T cell immune response potential
Source: Sci Rep. 2024 Jul 19;14:16721. doi: 10.1038/s41598-024-67099-2 (PMC11271619; doi:10.1038/s41598-024-67099-2)
Supplement: Supplementary file 1 — Supplementary Information. [file 41598_2024_67099_MOESM1_ESM.docx]

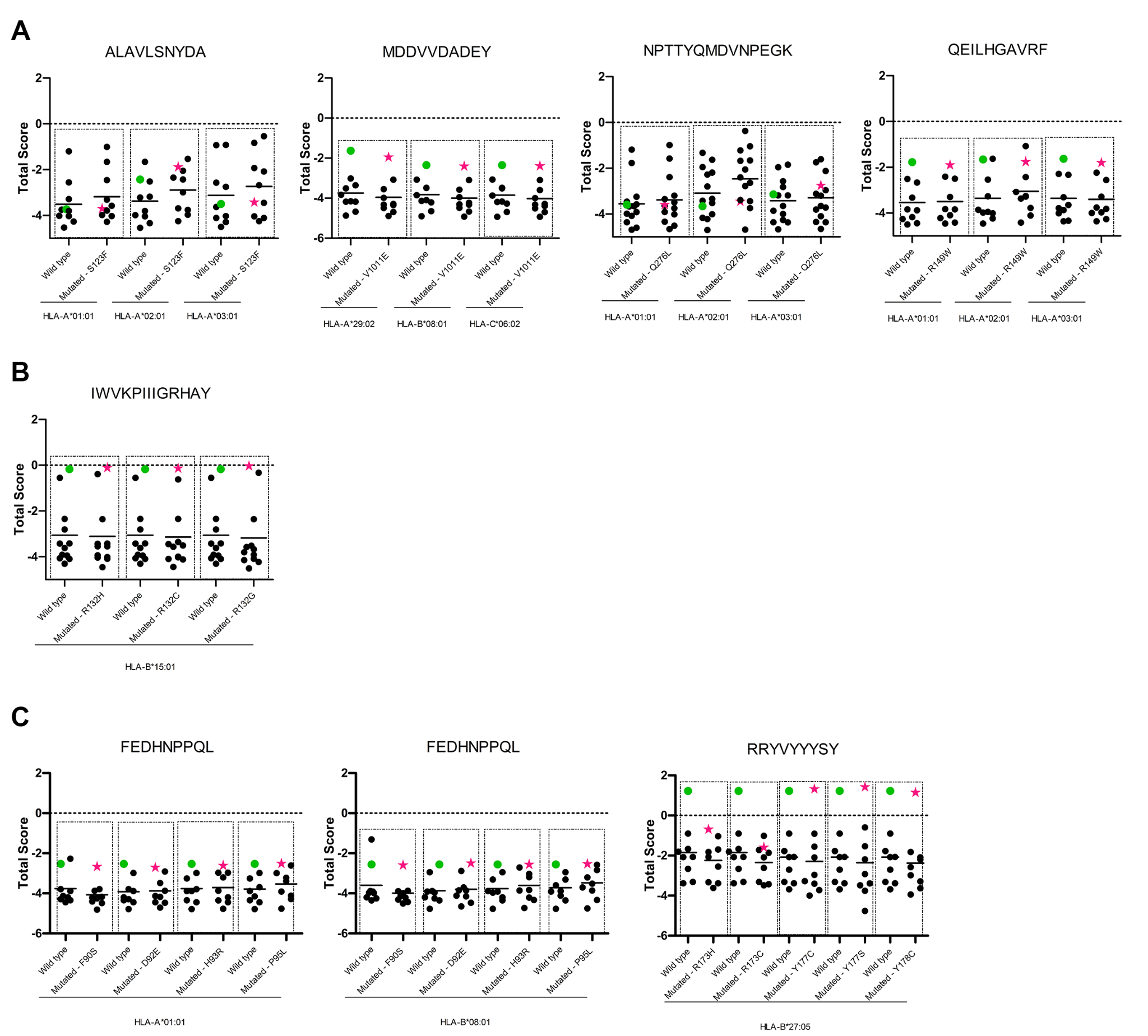


Supplementary Figure 1. The graphs illustrate the average total scores for epitopes originating from both wild-type and mutated (A) EGFR, (B) IDH1, and (C) PTEN proteins, as analyzed with the IEDB tool. In these graphs, green dots represent wild-type epitopes, pink dots denote mutated epitopes, and black dots indicate selected epitopes adjacent to the mutated regions. The visualizations were created using GraphPad Prism software.


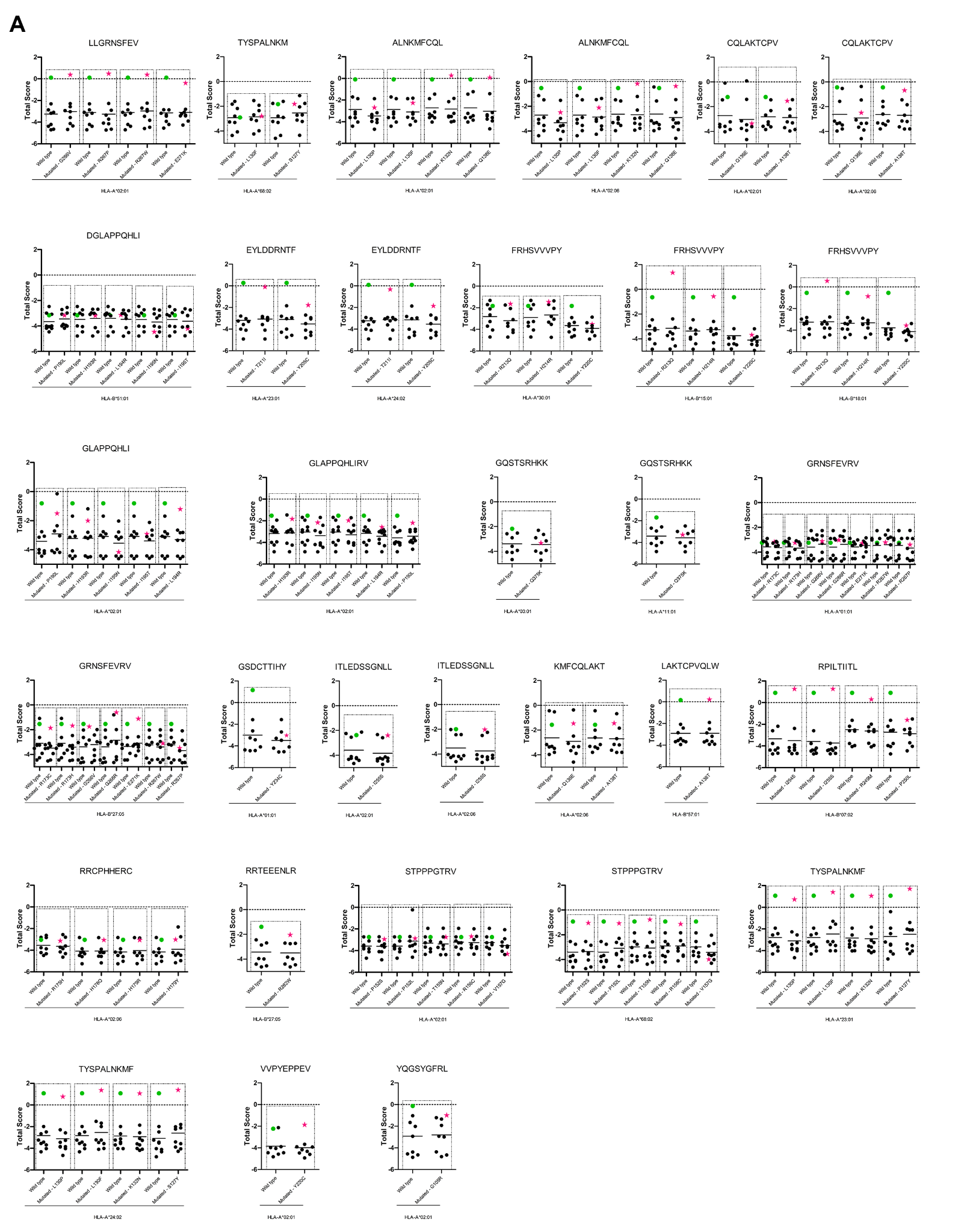


Supplementary Figure 2. The graphs illustrate the average total scores for epitopes originating from both wild-type and mutated (A) TP53 protein, as analyzed with the IEDB tool. In these graphs, green dots represent wild-type epitopes, pink dots denote mutated epitopes, and black dots indicate selected epitopes adjacent to the mutated regions. The visualizations were created using GraphPad Prism software.


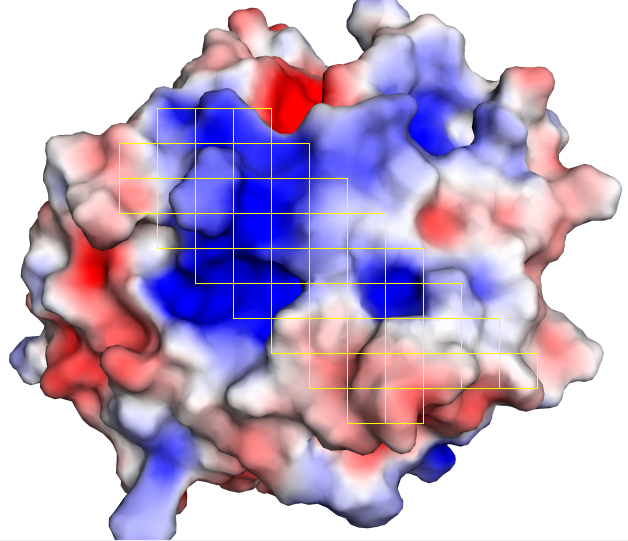


**Supplementary Figure**  **3. Top view of a pMHC-I complex (i.e., TCR-interacting surface) colored according to electrostatic potential (red, white, and blue represent negative, neutral, and positive charges, respectively). We used ImageJ tool to extract RGB mean and standard deviation values from the region defined by the 46 yellow squares (https://imagej.nih.gov/ij/).** The electrostatic potential range used was from -3kT to +3kT.

**
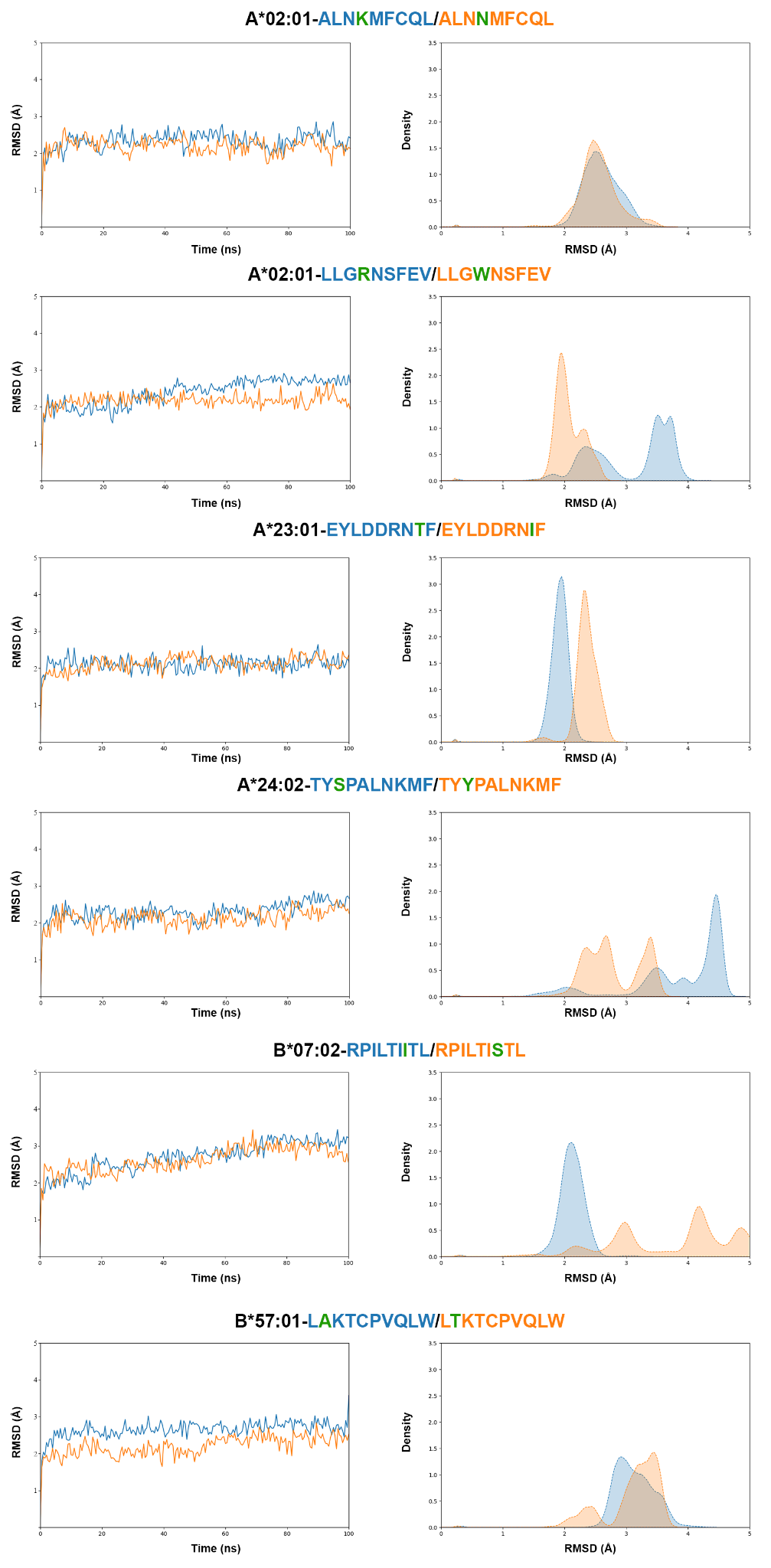
**

**Supplementary Figure 4.** Epitope RMSD (in Å) for 6 selected pMHC-I pairs. The graphs in the left shows the RMSD along time (in nanoseconds), while the graphs in the right shows the RMSD density plot for the whole simulation. Wild-type peptides are colored in blue, while the mutated counterpart is colored in orange. The mutated residue is shown in green.


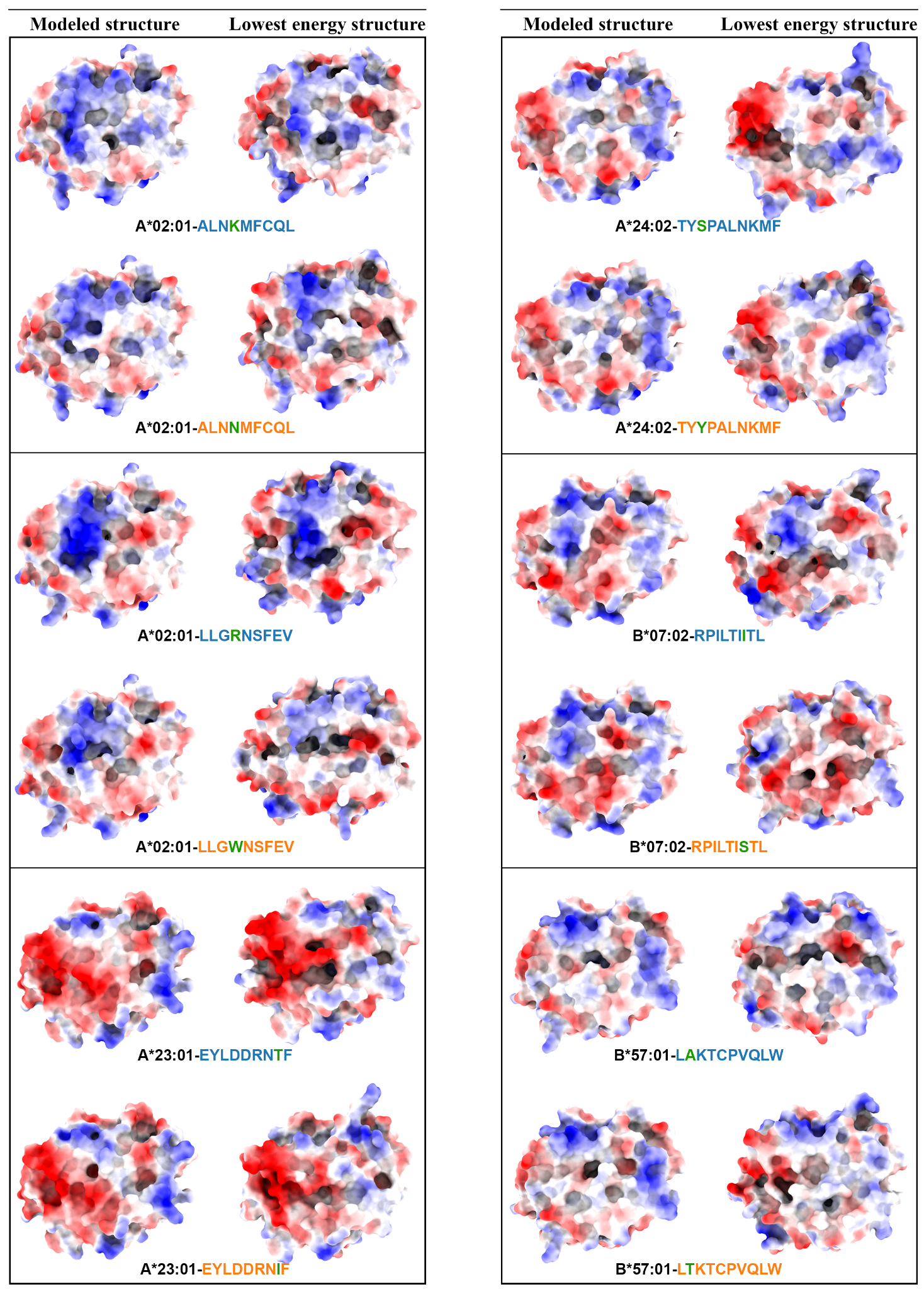


**Supplementary Figure 5. Comparison between the modeled pMHC-I and the lowest energy structure retrieved from the MD simulation for each complex.**

| **EGFR** | **IDH1** | **PTEN** | **TP53** |
| --- | --- | --- | --- |
| A289V | R132H | G132D | R175H |
| G598V | R132C | R130Q | R248Q |
| R222C | Y319F | C71Y | R248W |
| A289D | R132G | P246L | R282W |
| V774M | G300C | T277I | Y220C |
| A289T |  | C136Y | R273C |
| R108K |  | T131A | G245S |
| R252C |  | N276S | V216M |
| T363I |  | H272P | R158H |
| H304Y |  | G129V | R273H |
| T263P |  | G36E | H179R |
| S645C |  | R159K | C275Y |
| Y270C |  | G230E | D281H |
| L62R |  | R74T | T155N |
| G63R |  | G129E | C238Y |
| R149W |  | W274C | D281A |
| V651M |  | M270I | Y234C |
| D256G |  | H123Y | C238F |
| L861Q |  | L70H | R156C |
| C628Y |  | D107Y | Q136E |
| S380F |  | D109N | V157G |
| P596R |  | R11I | S362T |
| C187Y |  | F195I | I254S |
| C636Y |  | P204L | P152S |
| G598A |  | F271S | I255S |
| G63E |  | R159S | G266V |
| S229C |  | C105R | H193R |
| C620W |  | M239R | T211I |
| V1011E |  | C136R | K164E |
| R675W |  | A126V | R249M |
| P596S |  | W274S | R249T |
| D256Y |  | R173H | L130P |
| Q276L |  | R173C | H179Y |
| F254I |  | G36R | R248L |
| R677H |  | S170N | F328I |
| C624F |  | G165E | Q375K |
| C571S |  | Y178C | I195N |
| G503V |  | V45E | P250L |
| R252P |  | Y46H | R267P |
| P154H |  | Y27C | S241P |
| R108G |  | D92E | G244D |
| C240Y |  | C105Y | G244S |
| C620Y |  | V85F | K132N |
| C628F |  | I101T | P152L |
| W608C |  | L42V | V172G |
| R334C |  | R15K | S241Y |
| S123F |  | D107H | E271K |
| R1068Q |  | Y177C | L194R |
| D460E |  | L25V | Y205C |
|  |  | Y88H | G105R |
|  |  | G44D | R267W |
|  |  | Y177S | H214R |
|  |  | H93R | A138T |
|  |  | F90S | R213Q |
|  |  | T167S | R158P |
|  |  | P95L | A307V |
|  |  | R47K | M246R |
|  |  |  | I195T |
|  |  |  | P190L |
|  |  |  | G266R |
|  |  |  | H178Q |
|  |  |  | L130F |
|  |  |  | C242Y |
|  |  |  | S127Y |

**Supplementary Table 1.** Identification of *missense* mutations available for EGFR, IDH1, PTEN, and TP53 proteins, obtained from the TCGA-GDC cancer database (https://portal.gdc.cancer.gov/).

| **Protein** | **Epitope sequence  (IEDB)** | **Start** | **End** | **MHC Allele** | **Mutation (GDC)** |
| --- | --- | --- | --- | --- | --- |
| EGFR | MDDVVDADEY | 1007 | 1016 | HLA-A*29:02 HLA-B*08:01 HLA-C*06:02 | V1011E |
|  | ALAVLSNYDA | 118 | 127 | HLA-A*02:01 HLA-A*03:01 HLA-A*01:01 | S123F |
|  | NPTTYQMDVNPEGK | 271 | 284 | HLA-A*02:01 HLA-A*03:01 HLA-A*01:01 | Q276L |
|  | QEILHGAVRF | 141 | 150 | HLA-A*02:01 HLA-A*03:01 HLA-A*01:01 | R149W |
| IDH1 | WVKPIIIGRHAY | 124 | 135 | HLA-B*15:01 | R132H R132C R132G |
| PTEN | FEDHNPPQL | 90 | 98 | HLA-A*01:01 HLA-B*08:01 | F90S D92E H93R P95L |
|  | RRYVYYYSY | 172 | 180 | HLA-B*27:05 | R173H R173C  Y177C Y177S Y178C |
| TP53 | GRNSFEVRV | 266 | 274 | HLA-B*27:05 HLA-A*01:01 | R273C R273H G266V G266R E271K R267W R267P |
|  | TYSPALNKMF | 125 | 134 | HLA-A*24:02 HLA-A*23:01 | L130P  L130F  K132N S127Y |
|  | GLAPPQHLIRV | 187 | 197 | HLA-A*02:01 | H193R I195N I195T L194R P190L |
|  | RPILTIITL | 249 | 257 | HLA-B*07:02 | I254S I255S R249M P250L |
|  | LLGRNSFEV | 264 | 272 | HLA-A*02:01 | G266V R267P R267W E271K |
|  | GSDCTTIHY | 226 | 234 | HLA-A*01:01 | Y234C |
|  | EYLDDRNTF | 204 | 212 | HLA-A*23:01 HLA-A*24:02 | T211I Y205C |
|  | VVPYEPPEV | 217 | 225 | HLA-A*02:01 | Y220C |
|  | TYSPALNKM | 125 | 133 | HLA-A*68:02 | L130F S127Y |
|  | ALNKMFCQL | 129 | 137 | HLA-A*02:06 HLA-A*02:01 | Q136E  L130P L130F K132N |
|  | STPPPGTRV | 149 | 157 | HLA-A*68:02 HLA-A*02:01 | T155N R156C V157G P152S P152L |
|  | YQGSYGFRL | 103 | 111 | HLA-A*02:01 | G105R |
|  | FRHSVVVPY | 212 | 220 | HLA-B*15:01 HLA-A*30:01 | Y220C H214R R213Q |
|  | GQSTSRHKK | 374 | 382 | HLA-A*03:01 HLA-A*11:01 | Q375K |
|  | LAKTCPVQLW | 137 | 146 | HLA-B*57:01 | A138T |
|  | DGLAPPQHLI | 186 | 195 | HLA-B*51:01 | H193R I195N I195T L194R P190L |
|  | CQLAKTCPV | 135 | 143 | HLA-A*02:01 | Q136E A138T |
|  | ITLEDSSGNLL | 255 | 265 | HLA-A*02:01 | I255S |
|  | KMFCQLAKT | 132 | 140 | HLA-A*02:06 | Q136E A138T |
|  | RRCPHHERC | 174 | 182 | HLA-A*02:06 | R175H H179R H179Y H178Q |
|  | GLAPPQHLI | 187 | 195 | HLA-A*02:01 | H193R I195N I195T P190L L194R |
|  | RRTEEENLR | 282 | 290 | HLA-B*27:05 | R282W |

**Supplementary Table 2.** Immunogenic epitopes identified in the Immune Epitope Database (IEDB) and their corresponding MHC presentation molecules. 'Start' and 'End' denote the positions of the first and last amino acids of each epitope within the full protein sequence. The mutation found in GDC is also shown.

| **Protein** | **HLA (allele)** | **MHCflurry  Cutoff (nM)** | **Wild-type** | **Mutated** |
| --- | --- | --- | --- | --- |
| EGFR | A*29:02 | 3853 | MDDVVDADEY | MDDVEDADEY - V1011E |
|  | C*06:02 | 35121 | MDDVVDADEY | MDDVEDADEY - V1011E |
|  | A*03:01 | 18472 | NPTTYQMDVNPEGK | NPTTYLMDVNPEGK - Q276L |
|  | A*01:01 | 24050 | QEILHGAVRF | QEILHGAVWF - R149W |
|  | A*02:01 | 21184 | QEILHGAVRF | QEILHGAVWF - R149W |
| PTEN | A*01:01 | 19691 | FEDHNPPQL | FEEHNPPQL - D92E |
|  |  |  |  | SEDHNPPQL - F90S |
|  |  |  |  | FEDRNPPQL - H93R |
|  |  |  |  | FEDHNLPQL - P95L |
|  | B*08:01 | 21805 | FEDHNPPQL | SEDHNPPQL - F90S |
|  |  |  |  | FEDHNLPQL - P95L |
|  | B*27:05 | 27 | RRYVYYYSY | RCYVYYYSY - R173C |
|  |  |  |  | RRYVYCYSY - Y177C |
|  |  |  |  | RRYVYSYSY - Y177S |
|  |  |  |  | RRYVYYCSY - Y178C |
| TP53 | A*01:01 | 23397 | GRNSFEVRV | GRNSFEVHV - R273H |
|  |  |  |  | GRNSFEVCV - R273C |
|  |  |  |  | GWNSFEVRV - R267W |
|  |  |  |  | GPNSFEVRV - R267P |
|  |  |  |  | GRNSFKVRV - E271K |
|  | B*27:05 | 385 | GRNSFEVRV | VRNSFEVRV - G266V |
|  |  |  |  | GRNSFKVRV - E271K |
|  | A*23:01 | 18 | TYSPALNKMF | TYSPAPNKMF - L130P |
|  | A*24:02 | 39 | TYSPALNKMF | TYSPAPNKMF - L130P |
|  | A*02:01 | 172 | GLAPPQHLIRV | GLAPPQRLIRV - H193R |
|  | A*02:01 | 39 | LLGRNSFEV | LLGRNSFKV - E271K |
|  | A*24:02 | 64 | EYLDDRNTF | EYLDDRNIF - T211I |
|  | A*68:02 | 8440 | TYSPALNKM | TYYPALNKM - S127Y |
|  | A*02:06 | 324 | ALNKMFCQL | ALNKMFCEL - Q136E |
|  | A*02:01 | 9506 | STPPPGTRV | STPLPGTRV - P152L |
|  |  |  |  | STPSPGTRV - P152S |
|  |  |  |  | STPPPGTCV - R156C |
|  |  |  |  | STPPPGNRV - T155N |
|  | A*68:02 | 21 | STPPPGTRV | STPLPGTRV - P152L |
|  |  |  |  | STPSPGTRV - P152S |
|  |  |  |  | STPPPGTCV - R156C |
|  | A*30:01 | 7438 | FRHSVVVPY | FQHSVVVPY - R213Q |
|  | B*15:01 | 385 | FRHSVVVPY | FRRSVVVPY - H214R |
|  | B*51:01 | 752 | DGLAPPQHLI | DGLAPPQRLI - H193R |
|  |  |  |  | DGLAPPQHRI - L194R |
|  | A*02:01 | 321 | CQLAKTCPV | CQLTKTCPV - A138T |
|  | A*02:06 | 35 | CQLAKTCPV | CQLTKTCPV - A138T |
|  | A*02:01 | 5712 | ITLEDSSGNLL | STLEDSSGNLL - I255S |
|  | A*02:06 | 553 | ITLEDSSGNLL | STLEDSSGNLL - I255S |
|  | A*02:06 | 119 | KMFCQLAKT | KMFCQLTKT - A138T |
|  | A*02:06 | 29179 | RRCPHHERC | RRCPQHERC - H178Q |
|  |  |  |  | RHCPHHERC - R175H |

**Supplementary Table 3.** Filtered peptides with the same cutoff value of the predicted for wild-type peptide on MHCflurry for the different alleles (not obtained the structural image). The mutated amino acids are highlighted (missense) according to the GDC-Cancer.

|  |  |  |  |  |
| --- | --- | --- | --- | --- |
|  |  |  |  |  |
|  |  |  |  |  |
|  |  |  |  |  |
|  |  |  |  |  |
|  |  |  |  |  |
|  |  |  |  |  |
